# Mirror, mirror on the wall: which microbiomes will help heal them all?

**DOI:** 10.1186/s12916-016-0622-6

**Published:** 2016-05-04

**Authors:** Renuka R. Nayak, Peter J. Turnbaugh

**Affiliations:** Department of Microbiology and Immunology, G.W. Hooper Foundation, University of California San Francisco, 513 Parnassus Avenue, San Francisco, CA 94143 USA

**Keywords:** Gut microbiome, Pharmacology, Genetics, Precision medicine, Pharmaco-metagenomics

## Abstract

**Background:**

Clinicians have known for centuries that there is substantial variability between patients in their response to medications—some individuals exhibit a miraculous recovery while others fail to respond at all. Still others experience dangerous side effects. The hunt for the factors responsible for this variation has been aided by the ability to sequence the human genome, but this just provides part of the picture. Here, we discuss the emerging field of study focused on the human microbiome and how it may help to better predict drug response and improve the treatment of human disease.

**Discussion:**

Various clinical disciplines characterize drug response using either continuous or categorical descriptors that are then correlated to environmental and genetic risk factors. However, these approaches typically ignore the microbiome, which can directly metabolize drugs into downstream metabolites with altered activity, clearance, and/or toxicity. Variations in the ability of each individual’s microbiome to metabolize drugs may be an underappreciated source of differences in clinical response. Complementary studies in humans and animal models are necessary to elucidate the mechanisms responsible and to test the feasibility of identifying microbiome-based biomarkers of treatment outcomes.

**Summary:**

We propose that the predictive power of genetic testing could be improved by taking a more comprehensive view of human genetics that encompasses our human and microbial genomes. Furthermore, unlike the human genome, the microbiome is rapidly altered by diet, pharmaceuticals, and other interventions, providing the potential to improve patient care by re-shaping our associated microbial communities.

## Background

**Fig. 1:**
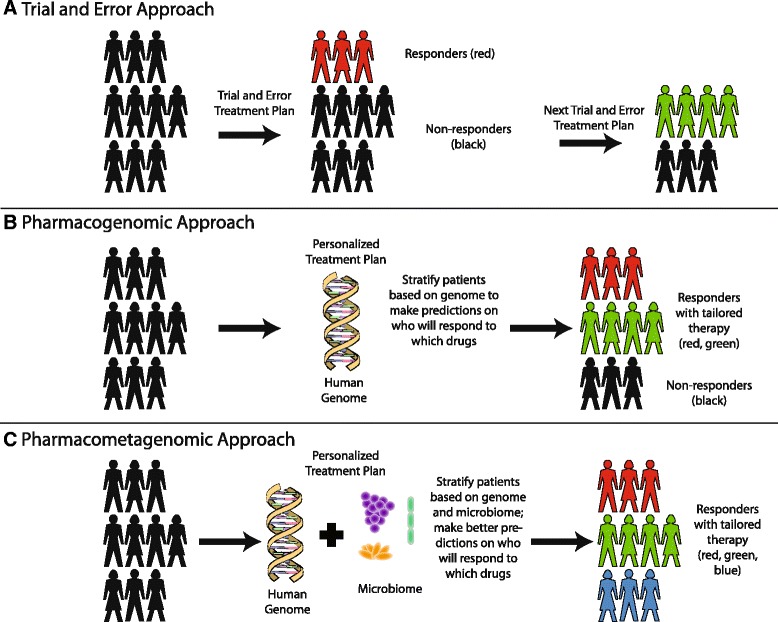
A vision for the future: knowledge of the microbiome can lead to better predictions of drug response. **a** Currently, most medications are prescribed in a trial-and-error fashion. It has been estimated that only 30–65 % of patients respond to most drugs [5]. Non-responders need to undergo iterative rounds of trial-and-error treatments before physicians and patients arrive at an adequate drug regimen that treats disease. **b** Human genome sequencing has enabled physicians to predict responses to medication based on host genotype. However, this is routine clinical practice for only a few drugs and there is still considerable room to improve our predictions. **c** We envision a future where combined information from a person’s genome (or epigenome, proteome, metabolome) and microbiome will be used to predict the best treatment for patients. These predictions will enable tailored therapy that reduces the amount of time that patients suffer and likelihood of developing adverse effects from therapy

The concept of “precision medicine” is a tantalizing possibility. Advances in sequencing the human genome led to the hypothesis that genetic differences may explain the incredible variation that clinicians observe when treating patients (Fig. 1a) [1]. If successful, this area of study would answer long-standing scientific questions with immediate translational implications: why do some patients respond to a particular treatment whereas others experience no benefit whatsoever? Why do some patients incur life-threatening reactions to drugs whereas others barely experience any side effects? Is it possible to predict these differences prior to initiating treatment instead of relying on patient observations and careful monitoring? Are there really one-size-fits-all treatment regimens or does every drug (and drug combination) need to be optimized for a given patient?

Multiple examples of the benefits of precision medicine are beginning to emerge (Fig. 1b). For example, several studies in HIV patients have suggested that routine testing for the HLA-B*5701 genotype prior to starting the anti-retroviral medication abacavir can lead to a reduction in severe hypersensitivity reactions to this drug [2]. Additionally, patients of Chinese and Thai descent undergo routine genetic testing for HLA-B*5801 prior to receiving allopurinol for gout, an inflammatory arthritis caused by urate crystals [3]. Patients with this locus exhibit severe skin, liver, and kidney reactions when given allopurinol, and thus these patients are instead treated with febuxostat.

Cancer therapeutics is another field where genetic testing has enabled tailored therapy. Patients with advanced cutaneous melanoma routinely have their tumors tested for the presence of a cancer-driving BRAF mutation, which is present in 40–60 % of patients [4]. Patients harboring the mutation are then successfully treated with vemurafenib or dabrafenib, which are inhibitors of BRAF [4].

Many other pharmacogenetic associations have been discovered, but are not routinely used clinically. In some cases, this is because there are limited studies demonstrating an improvement in care or because the genetic test is not cost effective [5]. This is true for drugs like warfarin and clopidogrel, which have been shown to be metabolized by the hepatic cytochrome P450 (CYP) enzymes CYP2C9 and CYP2C19, respectively [6]. While associations between these drugs and the CYP class of enzymes have been found, follow-up studies in patients have not convincingly shown that testing for these genes leads to better clinical outcomes [6].

Thus, for the vast majority of diseases, we are still far from tailoring the drug or dosage to a given patient’s genome [7]. In clinical practice, there are over 3,500 drugs in the US formulary, but only 35 drugs (<1 %) can be dosed based on genetic information [8, 9]. This number will undoubtedly increase with more advanced pharmacogenomic research; however, the human genome is just part of the picture. The microbiome, which is the collection of microbes (and their genes) that live in and on our bodies, also plays a role. If we take a more comprehensive view of our genome that includes our microbiome, the genetic variants in our human cells only account for a small part of the genetic differences observed between patients. Current estimates suggest that the number of unique genes found in the human microbiome outnumbers the human genome by multiple orders of magnitude [10]. Furthermore, while only ~1 % of the nucleotides found in the human genome vary between individuals [11], the microbiome is highly individualized [12]. Even identical twins raised together may only share 50 % of their gut bacterial species [13], and each bacterial species exhibits substantial copy number variation between individuals [14]. In adults, current estimates suggest that the majority of gut bacterial species can stably colonize each individual for years [15]. Importantly, these microbes are not passive bystanders; their genomes encode gene families that extend human metabolism by enabling the degradation of otherwise indigestible plant polysaccharides [16], the synthesis of essential vitamins and amino acids [17], and the biotransformation of xenobiotics (foreign compounds including drugs and dietary bioactive compounds) [18]. In this commentary, we discuss some of the emerging evidence demonstrating an important role for the gut microbiome in determining treatment success, the underlying mechanisms responsible, and the need for translational research strategies to begin to integrate these findings into clinical practice.

## Discussion

### Defining responders and non-responders

While many investigators have looked at the role of the microbiome in disease, more studies are needed to understand the contribution of the microbiome to variability in clinical response. There is substantial variation among patients in their response to treatment; one estimate suggests that most major drugs are effective in only 25–60 % of patients, with failures attributed to lack of efficacy or intolerable side effects [5]. Of clinical trials that are terminated, ~33 % are due to hepatotoxicity [19]. Some of this variation in drug response among patients has been shown to be from host genetic factors [20], but there is still considerable remaining variation that could be due to environmental factors and/or the microbiome. For example, one study examined variation in cholesterol levels and looked at the contributions of age, sex, genetics (human single nucleotide polymorphisms, or SNPs), and microbiome composition [21]. They found that the microbiome explained 4–6 % of the variation in cholesterol levels, and this was similar in magnitude to that explained by host genetics (between 3–7 %). This finding may suggest that diet shapes both the microbiome and cholesterol in a consistent way, or alternatively that the impact of the diet on cholesterol is mediated in part by the microbiome. Additional studies are necessary to elucidate these causal links.

The current clinical guidelines for evaluating drug response, despite their imperfections, are valuable for identifying which patients need more aggressive treatment and for setting general approaches to research the molecular underpinnings driving clinical variability. Drug response can either be measured as a continuous variable (e.g. disease activity index) or discrete categories (e.g. complete or partial response). The utility of characterizing patient response in this way is that it allows researchers to identify sub-populations warranting further study of the determinants of drug response.

Within the field of rheumatology, rheumatoid arthritis patients are monitored every three months to assess whether their disease is adequately controlled on their current drug regimen. If the clinical disease activity index (CDAI), a composite score of swollen and tender joints along with physician and patient rankings ranging from 0–76, is too high, then treatment is escalated [22]. However, rheumatologists currently lack a way to predict which medications will be most beneficial to the patient, and thus, treatment proceeds in a trial-and-error fashion (Fig. 1a). A major drawback is that precious time is lost in controlling disease and continued inflammation leads to worsening joint destruction.

Similarly, oncology patients would benefit from tailored treatment that would reduce the number of side effects and increase drug efficacy. Cancer treatment aims for “complete response” (i.e. no evidence of cancer), but sometimes patients can only achieve partial or no response while on a particular therapeutic regimen. Molecular medicine has facilitated greater tailoring of medications for oncology patients, but much work remains to be done [23].

Thus, one way to maximize the clinical utility of microbiome studies would be to quantify response to therapy. By using response criteria, investigators can then correlate treatment outcomes with changes in the microbiome. These associations can then be used to identify microbiome biomarkers that aid in predicting the most appropriate clinical strategy.

### Evidence that the microbiome can affect drug response

When drugs are taken orally, they are first exposed to our gut microbiome and can be modified before entering the human bloodstream [18]. In addition to controlling drug bioavailability, the gut microbiome can have multiple impacts on treatment response (Table 1). Gut microbial drug metabolism can produce downstream metabolites with decreased or increased efficacy. For example, the gut Actinobacterium *Eggerthella lenta* converts the cardiac drug digoxin into the downstream inactive microbial metabolite dihydrodigoxin [24]. In contrast, some microbes are necessary to produce the active compound. For example, sulfasalazine is hydrolyzed by gut bacterial azoreductases to 5-ASA and sulfapyridine. For inflammatory bowel disease, 5-ASA is thought to be the main active compound, whereas sulfapyridine is considered more important for rheumatoid arthritis [25]. To complicate things further, the parent drug sulfasalazine can inhibit the NFκB pathway whereas sulfapyridine cannot [26]. This example illustrates how the parent drug and its bacterial metabolites can have different mechanisms of action and presumably different targets. Microbial metabolism can also change drug clearance. For example, irinotecan is an anti-cancer drug that is converted into its active form SN-38. SN-38 is glucuronidated in the liver, aiding in its fecal excretion [27]. However, bacterial enzymes remove the glucuronide moiety from SN-38, effectively reactivating it and preventing its clearance. This reactivation in the gut also contributes to the dose-limiting diarrheal side effects of irinotecan [27]. Finally, the microbiome may mediate drug-drug interactions between antibiotics and other drugs [28]; for example, a recent study found that broad-spectrum antibiotics can diminish the microbial metabolism of lovastatin in rats [29].

**Table 1:** Direct impact of the gut microbiome on drug outcomes

Effect	Example	Microbial mechanism
Increased efficacy	Simvastatin [18]	Unknown.
Decreased efficacy	L-dopa [32, 33]	Exact mechanism is unknown, but it is suspected that *Helicobacter pylori* prevents duodenal absorption of L-dopa, directly metabolizes it, or binds it and prevents absorption.
Altered target specificity	Sulfasalazine [57]	Many gut bacteria possess azoreductases that cleave sulfasalazine into sulfapyridine and 5-ASA. The parent drug and its metabolites have different mechanisms of action and presumably different targets.
Increased clearance	Digoxin [24]	Proteins encoded by the *cgr* operon of *Eggerthella lenta* metabolize digoxin to an inactivate metabolite that is more readily excreted.
Decreased clearance	Pentobarbital [31]	Unknown gut microbes influence the abundance of liver enzymes that metabolize pentobarbital.
Increased toxicity	Irinotecan [27]	For irinotecan, multiple gut bacteria prevent clearance of SN-38 (the active metabolite of irinotecan) by removing a glucuronide group. This causes SN-38 to persist in the gastrointestinal tract and results in severe diarrhea.
Indirect interference with host metabolism	Acetaminophen [58]	For acetaminophen, multiple gut bacteria produce p-cresol, which competes with acetaminophen for drug clearance by liver enzymes.

In total, 50 drugs already have *in vitro* and/or *in vivo* evidence for metabolism by the gut microbiome [18]. More research is needed to determine if inter-individual differences in gut microbial community structure or function impact the outcome of these, and other, drugs. Comprehensive screens of microbes and drugs are necessary to determine the scope of gut microbial drug metabolism, as well as *in silico* approaches for predictive modeling. It may be useful to focus on drugs that have known variations in absorption, are administered orally, are subject to enterohepatic circulation, and/or are poorly soluble.

The gut microbiome may also indirectly affect how the host metabolizes or transports drugs. Comparisons of germ-free and colonized mice have revealed that gut microbes impact the expression of CYP enzymes in the liver, an essential enzyme family for drug detoxification [30, 31]. These differences in gene expression are functionally relevant; germ-free mice clear pentobarbital (an anesthetic) faster than colonized animals [31]. Gut bacteria can also affect transport of drugs across the gut lumen. For example, L-dopa, which is used to treat Parkinson’s, is bound by *Helicobacter pylori* and prevented from entering the bloodstream [32]. Treatment of *H. pylori* infection results in increased drug levels and efficacy of L-dopa in Parkinson’s patients [33].

It remains unclear why gut microbes have evolved mechanisms for manipulating the metabolism of foreign compounds like drugs [34]. One possibility is that enzymes that process related endogenous compounds have broad specificity—a type of “off-target” effect exacerbated by the vast metabolic potential encoded by the microbiome. Alternatively, it remains possible that even brief exposures to drugs can have significant effects on the fitness of gut microbes. Consistent with this hypothesis, multiple drugs target host enzymes and pathways that are also conserved in bacteria. For example, the anti-cancer drug 5-fluorouracil (5-FU) targets thymidylate synthase, a conserved enzyme necessary for DNA synthesis and cellular replication. In humans, this drug is inactivated by the enzyme dihydropyrimidine dehydrogenase (DPD). Bacteria also possess a version of DPD that is capable of inactivating 5-FU [35]. These results suggest that bacterial DPD may act on 5-FU before it reaches tumor tissue and that this microbial interaction may contribute to variability in treatment response among cancer patients.

Another example of functional redundancy between human and bacterial genomes is provided by the drug azathioprine, used in cancer and rheumatic diseases. The enzyme thiopurine methyltransferase (TPMT) is required to inactivate azathioprine. A small percentage of patients (<1 %) have mutations in TMPT that lead to reduced or complete loss of enzymatic activity—these patients suffer lethal side effects if given azathioprine [36]. Interestingly, TPMT is evolutionarily conserved and bacterial TPMT has activity against azathioprine [37]. Why would bacteria possess an enzyme to inactivate a cancer drug used to treat humans? Interestingly, in bacteria, this gene confers resistance to bactericidal drug tellurite [38], highlighting how bacterial enzymes can act promiscuously on drugs used to treat human disease. This provides another example of a bacterial enzyme that may inactivate a drug therapy before it reaches host tissue. Although physicians can screen patients for TPMT-inactivating mutations in the human genome prior to prescribing azathioprine, there is currently no test for the abundance or activity of TPMT in the microbiome.

Other pathways that may be targeted for metabolism by the microbiome are drugs that confer an evolutionary selective pressure, i.e. antibiotics. For example, metronidazole, a drug used to treat Crohn’s disease, has both anti-inflammatory and anti-microbial effects [39]. The inactivation of metronidazole by bacteria may be promoted by the selective pressure it places on the gut microbiome [18]. Even drugs that are not traditionally used as antibiotics can have antibacterial effects [40], such as omeprazole and sodium salicylate, the former of which has been shown to be metabolized by gut bacteria [18]. Indeed, recent studies demonstrate that the use of proton-pump inhibitors (PPIs) like omeprazole is associated with changes to the human gut microbiome [41, 42]. Thus, it is possible that when we use drugs with antimicrobial activity on patients to treat symptoms such as heartburn or pain, we are unintentionally altering the gut microbiome and selecting for microbes capable of drug metabolism.

### More research into the impact of the microbiome on drug response is needed

Numerous human microbiome studies have focused on correlating disease states to gut microbial community structure [43]. While valuable, these cross-sectional studies are challenging to interpret due to the many confounding factors found in patient populations, including the treatment itself [44] and the high degree of inter-individual variation in the gut microbiome [12]. Fortunately, many of these issues can be addressed by conducting intervention studies, where the collection of longitudinal data on the gut microbiome enables researchers to treat baseline samples from each individual as their own control. Yet, very few studies have examined associations between response to a therapeutic intervention and the gut microbiome.

One recent example comes from Kovatcheva-Datchary et al. [45], in which 39 human subjects were fed a barley kernel diet and blood glucose was examined. The responses, evaluated by postprandial blood glucose and insulin levels, varied markedly between individuals. Comparisons of the ten “most-responsive” to the ten “least-responsive” individuals revealed an increased abundance of the *Prevotella* genus in the top responders. Germ-free mice colonized with *Prevotella copri* demonstrated improved glucose metabolism compared to those colonized with heat-killed *P. copri* or *Bacteroides thetaiotaomicron*, providing causal evidence for the association identified in humans. Improved glucose homeostasis was also directly transmissible from responders to germ-free mice by colonizing them with responder stool samples, but not from non-responsive subjects. This study exemplifies the use of response criteria to identify and compare subjects in order to learn how the microbiome contributes to variability in treatment outcome. The investigators not only looked at correlation, but also examined causality, although the mechanisms by which *Prevotella* improves glucose metabolism have yet to be investigated.

Another way to identify the role of the microbiome in treatment response would be to collect and analyze stool samples from randomized controlled trials, which are the gold standard for inferring causality in humans. Doing so could lead to the identification of microbial consortia, individual microbes, genes, and/or metabolites that serve as biomarkers for treatment response. The identified organisms could then be further studied to determine genes or pathways that affect drug metabolism and confer varied clinical response. In the event that the trial fails to demonstrate a significant difference among treatment groups, post-hoc analyses can be used to identify whether the microbiome may contribute to drug efficacy. Then, more targeted clinical trials in which patients are sub-stratified based on their microbiomes may demonstrate a difference in treatment groups. In this way, clinically relevant aspects of the microbiome can be identified and targeted for further investigation and facilitate the success of clinical trials.

In addition to correlational studies, there is a need to examine the ways in which the microbiome plays a mechanistic role in pharmacology. We have yet to understand many of the bacterial species and genes involved in drug biotransformation and therapeutic response. Elucidating the molecular mechanisms responsible for microbial drug metabolism could permit the therapeutic targeting of microbial enzymes and opens up the possibility of microbiome engineering, an evolving research frontier in which microbes with synthetic pathways are constructed in order to carry out particular functions within an ecosystem [46].

Additional causal insights would need to come from germ-free, or gnotobiotic, mouse models with microbiomes that are derived from human donors [47]. These mice are referred to as “humanized”, and they enable studies of the human microbiome in a model organism in which numerous variables can be controlled in a way that cannot be ethically or logistically achieved when studying humans. These germ-free models also enable mono- or oligo-colonization with specific bacteria or bacterial consortia and allow researchers to determine whether specific bacteria confer disease phenotypes or affect drug metabolism.

### Learning about the microbiome has the potential to change clinical practice

While further investigation is clearly needed, there is tremendous potential to harness the microbiome to improve the treatment of human disease. The microbiome has the potential to predict who will respond to a particular intervention. Studies, such as those by Kovatcheva-Datchary et al. [45], demonstrate how the microbiome can contribute to human response to a dietary intervention and, thus, serve as both a biomarker and potential therapeutic target. It remains to be determined whether microbiome biomarkers are common or rare and whether they have large or small effect sizes. By comparison, most human genetic variants discovered thus far are rare with large effect sizes or common with weak effects [48].

Like the human genome, and many of the predictive SNPs that have been uncovered thus far, the microbiome does not need to be modified or causally linked to a phenotype of interest in order for it to be useful as a clinical biomarker. Features of the microbiome that can predict clinical response, either alone or in combination with host genetics, can be useful to physicians so long as the features are variable among patients, stable enough to be of predictive value, and better than pre-existing tools for predicting therapeutic efficacy. For example, baseline levels of the gut bacteria *Akkermansia muciniphila* have been shown to predict which patients have better nutritional parameters in response to a calorie-restricted diet [49]. While we have chosen to focus this commentary on the role of the microbiome in pharmacotherapy, there are now analogous examples of the predictive power of the microbiome in determining the success of nutritional interventions [50, 51].

A more mechanistic understanding of which microbes and which genes contribute to drug efficacy will enable a “pharmaco[meta]genomic” approach to precision medicine (Fig. 1c). Models encompassing genetics, epigenetics, and the microbiome may enable prediction of which patients will derive greatest benefit from a therapeutic intervention. For example, we have shown that digoxin is metabolized by select strains of *Eggerthella lenta*, and gut microbiomes with a higher abundance of the genes responsible for digoxin metabolism have a greater impact on drug levels [52]. Thus, a comprehensive understanding of which gut bacteria metabolize which drugs and the specific bacterial enzymes used for such biotransformations has the potential to change the way medications are prescribed to patients.

Furthermore, the ability to humanize gnotobiotic animals with a patient’s stool sample could allow investigators to test a particular intervention on a “humanized” animal before the intervention is carried out on the patient. This could allow for tailoring of therapies to each patient’s microbiome, allowing clinicians to empirically determine whether a patient will be a responder or not. Using these model systems, we can gain deeper understanding of how combinations of dietary, microbial, and pharmaceutical interventions act together to shape the recovery from disease.

In addition to acting as a predictive tool, the microbiome may be a valuable therapeutic target. Advances in genome editing [53] may soon enable the targeted deletion of microbial genes in clinical scenarios in which it is clear that treatment can be achieved with modification of a single process within the microbiome. The microbiome can also be readily modified by diet [54], antibiotics [55], or fecal transplantation [56].

## Summary

In conclusion, a deeper understanding of the human microbiome could lead to improvements in distinguishing responders versus non-responders, allowing physicians to provide precise, tailored treatment recommendations for their patients. Additional research is warranted to uncover the mechanisms through which gut microbes can contribute to a patient’s treatment success. Changes in the microbiome in response to therapy should be more broadly assessed in patient populations, perhaps through routine sampling of stool when conducting randomized controlled trials. Improved model systems, such as humanized mice, will be necessary to distinguish causal from casual associations and to develop more sophisticated approaches to analyzing and interpreting the human microbiome. If successful, these studies may soon begin to unlock the potential of the microbiome in serving as a predictive and therapeutic tool in clinical medicine.

## Abbreviations

5-FU: 5-fluorouracil
CDAI: clinical disease activity index
CYP: hepatic cytochrome P450
DPD: dihydropyrimidine dehydrogenase, PPI, proton-pump inhibitor
SNP: single nucleotide polymorphism
TPMT: thiopurine methyltransferase
